# Accelerating DNN Training Through Selective Localized Learning

**DOI:** 10.3389/fnins.2021.759807

**Published:** 2022-01-11

**Authors:** Sarada Krithivasan, Sanchari Sen, Swagath Venkataramani, Anand Raghunathan

**Affiliations:** ^1^Department of Electrical and Computer Engineering, Purdue University, West Lafayette, IN, United States; ^2^IBM Research, Yorktown Heights, NY, United States

**Keywords:** Deep Neural Networks (DNNs), localized learning, runtime efficiency, graphics process unit (GPU), stochastic gradient decent algorithm

## Abstract

Training Deep Neural Networks (DNNs) places immense compute requirements on the underlying hardware platforms, expending large amounts of time and energy. We propose LoCal+SGD, a new algorithmic approach to accelerate DNN training by *selectively combining localized or Hebbian learning within a Stochastic Gradient Descent (SGD) based training framework*. Back-propagation is a computationally expensive process that requires 2 Generalized Matrix Multiply (GEMM) operations to compute the error and weight gradients for each layer. We alleviate this by selectively updating some layers' weights using localized learning rules that require only 1 GEMM operation per layer. Further, since localized weight updates are performed during the forward pass itself, the layer activations for such layers do not need to be stored until the backward pass, resulting in a reduced memory footprint. Localized updates can substantially boost training speed, but need to be used judiciously in order to preserve accuracy and convergence. We address this challenge through a *Learning Mode Selection Algorithm*, which gradually selects and moves layers to localized learning as training progresses. Specifically, for each epoch, the algorithm identifies a *Localized*→*SGD* transition layer that delineates the network into two regions. Layers before the transition layer use localized updates, while the transition layer and later layers use gradient-based updates. We propose both static and dynamic approaches to the design of the learning mode selection algorithm. The static algorithm utilizes a pre-defined scheduler function to identify the position of the transition layer, while the dynamic algorithm analyzes the dynamics of the weight updates made to the transition layer to determine how the boundary between SGD and localized updates is shifted in future epochs. We also propose a low-cost weak supervision mechanism that controls the learning rate of localized updates based on the overall training loss. We applied LoCal+SGD to 8 image recognition CNNs (including ResNet50 and MobileNetV2) across 3 datasets (Cifar10, Cifar100, and ImageNet). Our measurements on an Nvidia GTX 1080Ti GPU demonstrate upto 1.5× improvement in end-to-end training time with ~0.5% loss in Top-1 classification accuracy.

## 1. Introduction

Deep Neural Networks (DNNs) have achieved continued success in many machine learning tasks involving images (Krizhevsky et al., 2017), videos (Ng et al., 2015), text (Zhou et al., 2015), and natural language (Goldberg and Hirst, 2017). However, training state-of-the-art DNN models is highly computationally expensive, often requiring exa-FLOPs of compute as the models are complex and need to be trained using large datasets. Despite rapid improvements in the capabilities of GPUs and the advent of specialized accelerators, training state-of-the-art models using current platforms is still quite expensive and often takes days to weeks. In this work, we aim to reduce the computational complexity of DNN training through a new algorithmic approach called LoCal+SGD^1^, which alleviates the key performance bottlenecks in Stochastic Gradient Descent (SGD) through *selective use of localized learning*.

**Computational Bottlenecks in DNN Training**. DNNs are trained in a supervised manner using gradient-descent based cost minimization techniques such as SGD (Bottou, 2010) or Adam (Kingma and Ba, 2015). The training inputs, typically grouped into minibatches, are iteratively forward propagated (*FP*) and back propagated (*BP*) through the DNN layers to compute weight updates that push the network parameters in the direction that decreases the overall classification loss. Back-propagation is computationally expensive, accounting for 65–75% of the total training time on GPUs. This is attributed to two key factors: (i) *BP* involves 2 Generalized Matrix Multiply (GEMM) operations per layer, one to propagate the error and the other to compute the weight gradients, and (ii) when training on distributed systems using data/model parallelism (Dean et al., 2012; Krizhevsky et al., 2012), aggregation of weight gradients/errors across devices incurs significant communication overhead.

**Prior Efforts on Efficient DNN Training**. Prior research efforts to improve DNN training time can be grouped into a few directions. One group of efforts enable larger scales of parallelism in DNN training through learning rate tuning (Goyal et al., 2017; You et al., 2017a,b) and asynchronous weight updates (Dean et al., 2012). Another class of efforts employ importance-based sample selection during training, wherein “easier” training samples are selectively discarded to improve runtime (Jiang et al., 2019; Zhang et al., 2019). Finally, model quantization (Sun et al., 2019) and pruning (Lym et al., 2019) can lead to significant runtime benefits during training by enabling the use of reduced-bitwidth processing elements.

LoCal+SGD**: Combining SGD with Localized Learning**. Complementary to the aforementioned efforts, we propose a new approach, LoCal+SGD, to alleviate the performance bottlenecks in DNN training, while preserving model accuracy. Our hybrid approach combines Hebbian or localized learning (Hebb, 1949) with SGD by selectively applying it in specific layers and epochs. Localized learning rules (Hebb, 1949; Oja, 1982; Zhong, 2005) utilize a single feed-forward weight update to learn the feature representations, eschewing the *BP* step. Careful formulation of the localized learning rule can result in substantial computation savings compared to SGD. Further, it also reduces memory footprint as activations from *FP* need not be retained until *BP*. The reduction in memory footprint can in turn allow increasing the batch size during training, which leads to further runtime savings due to better compute utilization and reduced communication costs. It is worth noting that localized learning has been extensively explored in the context of unsupervised learning (van den Oord et al., 2018; Hénaff et al., 2019; Chen et al., 2020). Further, the formulation of new neuro-inspired learning rules remains an active area of research (Lee et al., 2015; Nøkland, 2016). Our work is orthogonal to such efforts and represents a new application of localized learning in a fully supervised context, wherein we selectively employ it within an SGD framework to achieve computational savings.

Preserving model accuracy and convergence with LoCal+SGD requires localized updates to be applied judiciously i.e., only to selected layers in certain epochs. We address this challenge through the design of a *learning mode selection algorithm*. At the start training, the algorithm initializes the learning mode of all layers to SGD. As training progresses, it identifies layers that will be moved to localized learning. Specifically, for each epoch, the algorithm identifies a *Localized*→*SGD* transition layer, which delineates the network into two regions. Layers before the transition layer use localized updates, while subsequent layers use gradient-based updates. This allows *BP* to stop at the transition layer, as layers before it have no need for the back-propagated errors. We explore both static and dynamic learning mode selection algorithms. The static algorithm utilizes a suitably chosen pre-defined function to determine the position of the transition layer every epoch. The dynamic algorithm analyzes the dynamics of the weight updates of the *Localized*→*SGD* transition layer in deciding the new position of the boundary. Further, we provide weak supervision by modulating the learning rate of locally updated layers based on the overall training loss.

To the best of our knowledge, LoCal+SGD is the first effort that combines localized learning (an unsupervised learning technique) within a supervised SGD context to reduced computational costs while maintaining classification accuracy. Across 8 image recognition CNNs (including ResNet50 and MobileNet) and 3 datasets (Cifar10, Cifar100, and ImageNet), we demonstrate that LoCal+SGD achieves up to 1.5× improvement in training time with ~0.5% Top-1 accuracy loss on a Nvidia GTX 1080Ti GPU.

## 2. Materials and Methods: LoCal+SGD

The key idea in LoCal+SGD is to apply localized learning to selected layers and epochs during DNN training to reduce the overall training time with minimal loss in accuracy. The following design choices are critical to the effectiveness of LoCal+SGD:

- **Localized Learning Rule Formulation**. Eliminating *BP* would be of little help if it is replaced with an equally expensive learning rule. It is critical to choose a computationally efficient rule that still enables learning in the contexts where it is invoked.
- **Learning Mode Selection**. It is well known that universal use of localized learning rules results in an accuracy that is much lower than SGD. The key is to figure out when (which epochs) and where (which layers) to apply localized learning to best balance efficiency and accuracy. We refer to this as learning mode selection.
- **Weak Supervision**. Since we are operating within an overall supervised learning context where some layers are using global information, it is natural to ask whether such information can be used in a lightweight manner to improve the efficacy of localized learning. To this end, we propose a weak supervision technique, which modulates the learning rates of localized learning based on the overall classification loss.

In the following sub-sections, we describe how we address these design choices in greater detail.

### 2.1. Efficient Localized Learning

There has been growing interest toward the design of biologically plausible learning algorithms, in part to address the high computational requirements of stochastic gradient descent and in part to realize bio-plausible artificial intelligence systems. Learning rules such as feedback alignment Nøkland (2016) resolve the weight transport problem (Liao et al., 2015) by allowing for asymmetry in the weight values during forward and backward propagation. Similarly, target propagation (Lee et al., 2015) encourages neural activity to reach desired target activations evaluated during forward propagation, instead of utilizing loss gradients. Other learning rules such as equilibrium propagation (Scellier and Bengio, 2017) update the weights by evaluating gradients of locally defined objective functions, thereby avoiding gradient propagation across the network. However, many of these bio-plausible learning algorithms end up being computationally more expensive than SGD, such as feedback alignment (Nøkland, 2016). As the focus of our work is primarily on improving training runtime while achieving state-of-the-art accuracies, we propose the selective use of computationally lightweight localized learning rules in conjunction with SGD.

Localized learning has been extensively explored in the context of unsupervised learning, demonstrating success on small (< = 3 layer) networks using relatively simpler datasets (e.g., MNIST, Cifar-10) (Krizhevsky et al., 2009; Deng, 2012) with an accuracy gap that is yet to be bridged on larger datasets (e.g., ResNet50 or MobileNetV2 on ImageNet; Deng et al., 2009). First proposed in Hebb (1949), the key intuition behind localized learning rules is to encourage correlations between neurons that have similar activation patterns. Equation (1) depicts the Hebbian weight update proposed in Hebb (1949), for a synapse with weight *W*, connecting a pair of input and output neurons whose activation values are represented by *x* and *y*, respectively, with η as the learning rate.


(1) $$\begin{array}{r} △W=\eta \cdot x\cdot y \end{array}$$

Considerable research has gone into evolving this equation over the years to improve the performance of localized learning (Oja, 1982; Zhong, 2005). However, many of the proposed rules are computationally complex, or are difficult to parallelize on modern hardware platforms such as GPUs. Since our primary goal is improving DNN training time, we adopt the computationally simple localized learning rule presented in Equation (1).

**Figure 1:**
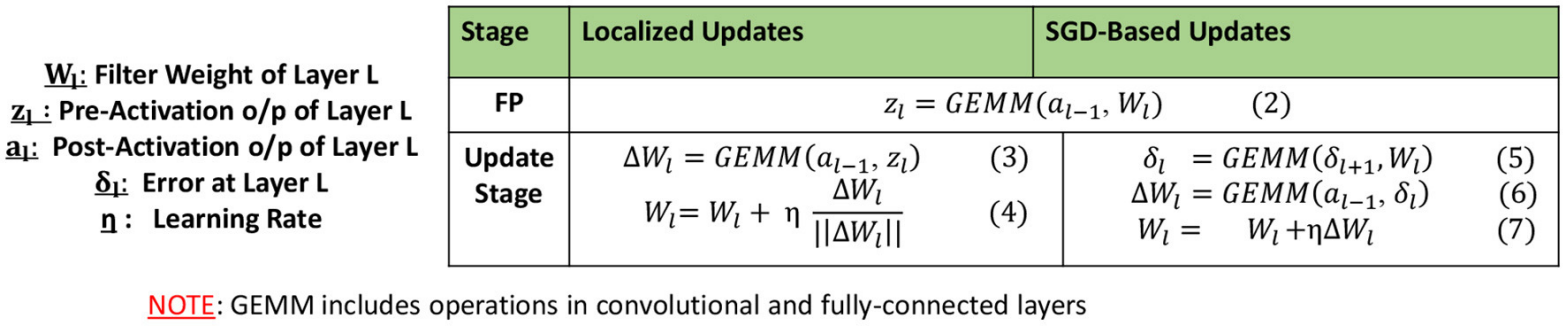
Comparing localized updates and SGD-based *BP*.

Note that the learning rule in Equation (1) assumes a distinct synapse between each input and output neuron pair. While its application to fully-connected (fc) layers is straightforward, we need to consider the sharing of weights between neuron pairs in convolutional (conv) layers. For updating a shared weight of a conv layer, we calculate the individual updates due to each pair of pre- and post-synaptic neurons sharing the weight and sum all such updates. This essentially reduces to a convolution operation between the input and output activations of the layer and can be expressed by Equation (3) in Figure 1. For further computational efficiency improvement, unlike Equation (1), we consider the pre-activation-function values of the outputs i.e., *z*_*l*_ instead of their post activation value *a*_*l*_. Further, we normalize the localized update values as shown in Equation (4) of Figure 1, as it was observed to achieve better convergence in practice.

Overall, we utilize Equations (3) and (4) from Figure 1 to perform the weight updates in all layers that are earlier than the *Localized*→*SGD* transition layer during a certain epoch. All other layers continue to be updated using SGD-based *BP*, expressed by Equations (5–7) in Figure 1. SGD updates are applied to batch-normalization layers present after the *Localized*→*SGD* transition layer, and are otherwise skipped. Clearly, Equation (3) has the same computational complexity as Equation (6) of SGD-based *BP* for conv and fc layers. Thus, from Figure 1, we can directly infer that our localized learning rule will be considerably faster than SGD-based *BP*. In practice, we measured this improvement to be more than 2× on a NVIDIA GTX 1080Ti GPU for the ImageNet-ResNet50 benchmark, across all conv and fc layers. In addition, localized learning also reduces the memory footprint of SGD-based *BP*. This is because DNN software frameworks commonly store all activation values computed during *FP* for use during SGD-based *BP* [*a*_*l*−1_ in Equation (6) of Figure 1]. In contrast, the localized update for a layer can be performed as soon as the *FP* through the layer is complete. The activation tensor *a*_*l*_ of layer *L* can be discarded or over-written as soon as *FP* proceeds to the next layer in the network, thereby freeing up a significant portion of on-device memory during training. In turn, this can allow larger minibatch sizes to be accommodated on a given hardware platform, when the localized updates are applied on a sufficient number of layers.

### 2.2. Learning Mode Selection Algorithm

The compute benefits of localized learning come at the cost of potential loss in accuracy with respect to SGD. To address this challenge, we propose a learning mode selection algorithm to judiciously choose when and where to apply localized learning. The algorithm identifies the learning mode of each layer in every epoch to create a favorable tradeoff between training time and accuracy.

Before describing the proposed learning mode selection algorithms, we first study the effects of different spatio-temporal patterns of localized learning on the computational efficiency and accuracy of a neural network. We specifically investigate whether localized learning is more suitable for specific layers in the network and specific phases in the training process.

*Impact on runtime*: We first analyze the impact of spatial patterns, i.e., whether applying localized learning to specific layers in the network results in better runtime. In a particular epoch, if a convolutional layer *L*, updated with SGD precedes a convolutional layer *K*, that is updated locally, calculating the SGD-based error gradients of Layer *L*, i.e., δ_*L*_, requires error propagation through the locally updated layer *K*. From a compute efficiency perspective, the benefits of using localized-updates in layer *K* vanish. Thus, it makes sense to partition the network into two regions—a prefix (set of initial layers) that are updated using localized learning, followed by layers that are updated with SGD. In such a setting, SGD-based *BP* is simply stopped at the junction of the two regions. Naturally, the compute benefits increase when the number of locally updated layers are higher and thus the boundary, which we refer to as the *Localized*→*SGD* transition layer, is moved deeper into the network.

The impact of different temporal patterns on runtime efficiency is quite straightforward, with higher number of locally updated epochs leading to proportionally higher benefits. Further, as the compute complexity of localized updates is constant across different epochs, these benefits are agnostic of the specific epochs in which localized learning is utilized.

*Impact on accuracy*: To analyze the impact on accuracy, we first examine the nature of features learnt by different layers trained by SGD. It is commonly accepted that the initial layers of a network perform feature extraction (Agrawal et al., 2014), while later layers aid in the classification process. As localized learning demonstrates better performance for feature extraction, applying it more aggressively, i.e., for higher number of epochs, in the initial layers has a much smaller impact accuracy. For later layers in the network, the number of localized learning epochs should be progressively reduced to preserve accuracy.

**Figure 2:**
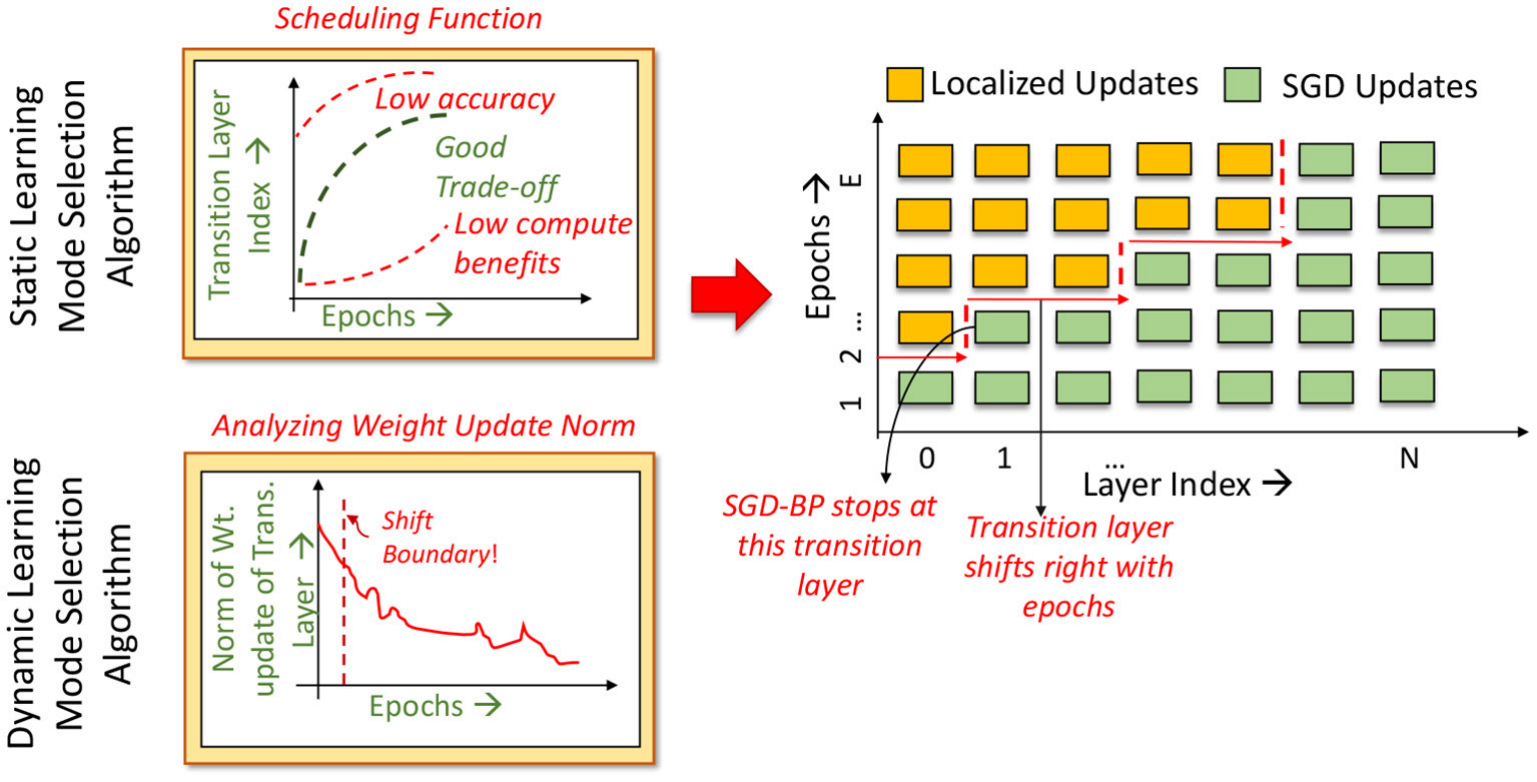
Overview of the learning mode selection algorithm.

Overall, based on the impact of localized learning on both runtime and accuracy, we find that a good learning mode selection algorithm should favor application of localized learning to a contiguous group of initial layers, while employing fewer localized learning epochs in later layers. We impose an additional constraint in order to ensure stability and convergence of training. We allow each layer to transition from one learning mode to another at most once during the entire training process. We empirically observe that utilizing SGD as the initial learning mode allows the network to achieve a higher accuracy than utilizing localized learning as the initial mode. In other words, SGD provides a better initialization point for the parameters of all layers, and the subsequent use of localized updates enables the training to converge with good accuracy. Taken together, the aforementioned constraints imply that if a layer *L* switches from the SGD learning to localized learning at epoch *E*, layer *L* + 1 may switch at an epoch *E*′ >= *E*. This is depicted graphically in Figure 2, where the *Localized*→*SGD* transition layer must move toward the right in successive epochs.

**Figure 3:**
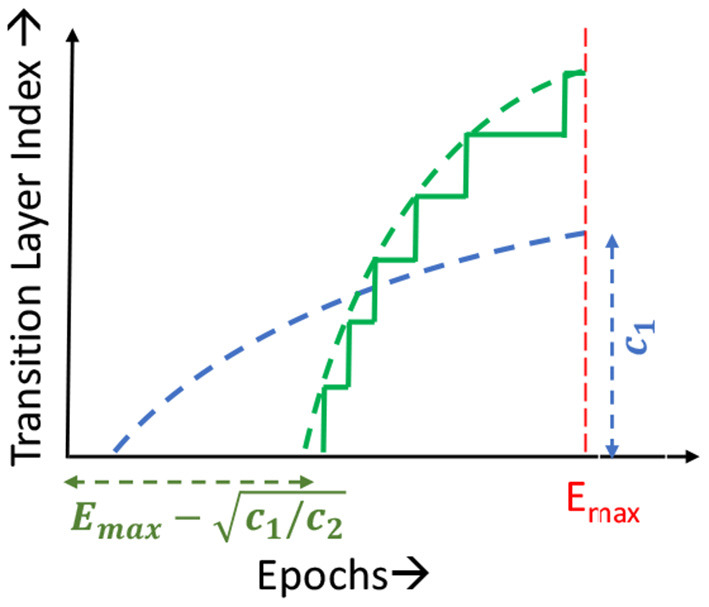
Transition layer schedules.

**Figure 4:**
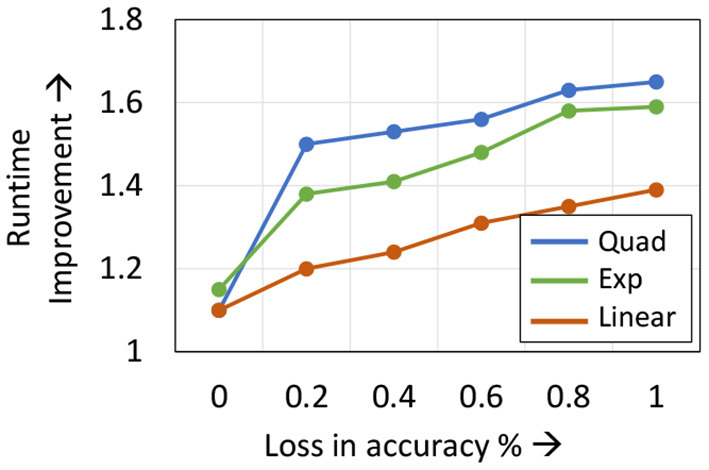
Impact of different scheduling functions on Cifar10-ResNet18 training.

**Static Learning Mode Selection Algorithm:** In a static learning mode selection algorithm, the *Localized*→*SGD* transition layer is computed using a pre-determined schedule (Figure 3). Many functions can be used to impose the desired schedule, wherein the number of locally updated layers increases monotonically with the epoch index. These functions must be chosen such that the schedule is neither too conservative in the application of localized updates (which may lead to sub-optimal compute and memory benefits), nor too aggressive (which may lead to a large drop in accuracy). In our experiments, we observed that using a quadratic function provides a good tradeoff between efficiency and accuracy. We illustrate this in Figure 4, wherein we compare the performance of quadratic, exponential and linear schedules for the Cifar10-ResNet18 benchmark. The proposed linear, quadratic and exponential scheduling functions that specifies the index of the *Localized*→*SGD* transition layer *N*_*l,E*_ at every epoch *E* are expressed as:


(2) $$\begin{array}{r} N=\left\lfloor max\left(c_{1}\cdot E_{max}+c_{2}\cdot E,0\right)\right\rfloor \end{array}$$

(3) $$\begin{array}{r} N=\left\lfloor max\left(c_{1}-c_{2}\cdot {\left(E-E_{max}\right)}^{2},0\right)\right\rfloor \end{array}$$

(4) $$\begin{array}{r} N=\left\lfloor max\left(\left(e^{c2\cdot E}-c_{1}\cdot E_{max}\right),0\right)\right\rfloor \end{array}$$

where *c*_1_ and *c*_2_ are hyper-parameters, and *E*_*max*_ is the total number of training epochs. As shown in Figure 3 for quadratic schedules, *c*_1_ controls the maximum number of layers that are updated locally across the training process, while *c*_2_ controls the epoch at which localized updates begin. The values of *c*_1_ and *c*_2_ are determined with the aim of maximizing the area under the curve, i.e., employing localized updates as many layers and epochs as possible, while maintaining a competitive classification accuracy.

**Dynamic Learning Mode Selection Algorithm:** As shown in Figure 4, the efficacy of the learning mode selection algorithm is dependent on the scheduling function chosen. Given the long training runtimes, identifying the optimal schedule for every network is a cumbersome process, and it is beneficial if the learning mode selection algorithm is free of hyper-parameters. To that end, we propose a dynamic learning mode selection algorithm that automatically identifies the position of the boundary every epoch.

The dynamic learning mode selection algorithm, described in Algorithm 1, analyzes the changes in the *L*_2_ norm of the SGD weight update of the *Localized*→*SGD* transition layer, and determines whether the boundary can be shifted deeper into the network for the next epoch. The exponentially running average of the norm update, *W*_*avg*_, is first evaluated (line 1). If the norm of the weight update in epoch *E* is significantly smaller than *W*_*avg*_, i.e., less than some fraction α, the boundary is shifted right by *L*_*shift*_ layers (line 2). Else, the boundary remains stationary (line 4). The rationale for this criterion is that sustained high magnitudes of SGD weight updates in the transition layer indicate that they are potentially critical to accuracy, in which case the transition layer must continue being updated with SGD.

**Algorithm 1:** Learning Mode Selection Algorithm.

**Input:** *T*_*E*_ (Index of the transition layer at epoch E), || △ *W*_*E*_|| (*L*_2_ norm of the weight update of the transition layer at epoch E), *L*_*shift*_ (number of layers to shift boundary)
**Output:** *T*_*E*+1_ (Index of the transition layer at epoch E+1)
1: **if** || △ *W*_*E*_|| < = α · *W*_*Avg*_
2: *T*_*E*+1_ = *T*_*E*_ + *L*_*shift*_
3: **else**
4: *T*_*E*+1_ = *T*_*E*_

Naturally, α and *L*_*shift*_ provide trade-offs between accuracy and runtime savings—higher values of either quantity result in aggressive applications of localized updates and hence better runtimes, but at the cost of degradations in accuracy. Our experiments suggest that values of α between 0.1 and 0.5, and *L*_*shift*_ between 10 and 15%, provide good performance across all the benchmarks studied. In section 3, we explore this trade-off space in greater detail.

To summarize, we propose static and dynamic learning mode selection algorithms that help identify the position of the transition layer for every epoch. Each algorithm comes with its own benefits—static algorithms can be hand-tuned to provide superior performance, but at the cost of additional effort involved in tuning the hyperparameters.

### 2.3. Weak Supervision

To further bridge the accuracy gap between our hybrid and end-to-end SGD training, we introduce weak supervision in the locally updated layers. Unlike the SGD, the localized learning rules described thus far do not take advantage of the information provided by supervision, i.e., the classification error evaluated at the output. We incorporate this information through a low-cost weak supervision scheme that consists of a single signal sent to all layers updated locally in a particular epoch. This feedback is derived from the classification loss observed over past few epochs. The weak supervision scheme is described in Algorithm 2.

**Algorithm 2:** Weak Supervision Scheme.

**Input:** *L*_*i*_ (Overall classification loss at epoch *i*), *lr*_*L*_ (original learning rate of layer *L*)
**Output:** *W*_*L*_ (Weight update of layer *L*)
1: △ *W*_*L*_ = *conv*(*a*_*l*−1_, *z*_*l*_)
2: **if** *L*_*i*−1_ < *L*_*i*_
3: *W*_*L*_ = *W*_*L*_ - *lr*_*L*_ · $\frac{△W_{L}}{\left||△W_{L}|\right|}$
4: **else**
5: *W*_*L*_ = *W*_*L*_ + *lr*_*L*_ · $\frac{△W_{L}}{\left||△W_{L}|\right|}$

The key principle behind the weak supervision scheme is to control the learning rates of the locally updated layers based on the rate at which the overall classification loss changes. For example, if the overall classification loss has increased across consecutive epochs, we reverse the direction of the updates (line 3) in the next epoch. In contrast, the update direction is maintained if the overall loss is decreasing (line 5). We find that this weak supervision provides better accuracy results than other learning rate modulation techniques for the locally updated layers such as Adam or momentum-based updates.

We would like to highlight that traditional SGD provides fine-grained supervision and involves evaluating the error gradients for every neuron in the network. In contrast, the proposed weak supervision scheme provides coarse-grained supervision by forcing all weights to re-use the same loss information. Overall, our weak supervision scheme is not developed with the intent to compete with SGD updates, but is rather a simple, approximate and low-cost technique that brings the final accuracy of LoCal+SGD closer to end-to-end SGD training.

## 3. Results and Discussion

In this section, we present the results of our experiments highlighting the compute benefits achieved by LoCal+SGD. We evaluate the benefits across a suite of 8 image-recognition DNNs across 3 datasets. We consider the ResNet18 (He et al., 2015) and VGG13 (Simonyan and Zisserman, 2015) networks for the Cifar10 (Krizhevsky et al., 2009) and Cifar100 (Krizhevsky et al., 2009) datasets; and the ResNet34, ResNet50 (He et al., 2015) and MobileNetV2 (Sandler et al., 2018) networks for the ImageNet dataset (Deng et al., 2009).

### 3.1. Experimental Setup

This subsection describes the experimental setup used for realizing the baseline and proposed LoCal+SGD training schemes. We conduct our experiments on the complete training and test datasets of each benchmark, using the PyTorch (Paszke et al., 2019) framework. All experiments are conducted on Nvidia GTX 1080Ti GPUs with the batch size set to 64 per GPU, unless otherwise mentioned.

**Baseline:** We consider end-to-end SGD training as the baseline in our experiments. The hyper-parameters used in SGD training of each of the benchmarks are described below.

ImageNet: For experiments in section 3.2 we utilize a batch-size of 64 per GPU, for all benchmarks. For the ResNet50 and ResNet34 benchmarks the initial learning rate set to 0.025. The learning rate is decreased by 0.1 every 30 epochs, for a total training duration of 90 epochs, and the weight decay is 4*e* − 5. The MobileNetV2 benchmark utilizes an initial learning rate of 0.0125. We use a cosine learning rate decay schedule, as in Li et al. (2019) for 150 epochs. The weight decay is set to 4*e* − 5. Both benchmarks use an input size of 224*224*3.

For the experiments in section 3.3, the total batch-size at epoch 1 is 256 (64*4), with the initial learning rate set to 0.1 for the ResNet benchmarks and 0.05 for the MobileNetV2 benchmark. All other parameters remain the same.

Cifar10 and Cifar100: All Cifar10 and Cifar100 experiments utilize a batch-size of 64. The Cifar10 benchmarks are trained with an initial learning rate of 0.05 that is decayed by 0.1 every 10 epochs, across 90 epochs. The initial learning rate of the Cifar100 benchmarks is 0.025 and decayed by 0.5 every 20 epochs, for 150 epochs in total. The weight decay is set to 5*e* − 4. Both benchmarks utilize an input size of 32*32*3.

LoCal+SGD: In the proposed LoCal+SGD training scheme, the layers updated with SGD are trained with the same hyper-parameters used in the baseline implementation. Further, LoCal+SGD training is conducted using the same number of epochs as baseline SGD training. When a layer is updated locally, the initial learning rate is 0.01 and is decayed by factors of 2 and 10 every 30 epochs for the Cifar and the ImageNet benchmarks, respectively. In all experiments, the α parameter is set to 0.8. We measure the accuracy and runtime of the proposed scheme for the same number of training epochs as the baseline implementations. Further, we utilize the same random seed to initialize the weights of the network when comparing the performance of LoCal+SGD against the baseline. Speed-up and accuracy results are averaged over 10 runs for each benchmark.

### 3.2. Single GPU Execution Time Benefits

**ImageNet:** Table 1 presents the performance of the baseline (end-to-end SGD training) and the proposed LoCal+SGD algorithm (both static and dynamic versions) on the ImageNet benchmarks in terms of the Top-1 classification error and runtime observed on a single GPU. For all benchmarks listed here, the static and dynamic versions of LoCal+SGD apply localized updates for nearly 50–60% of the layers. Further, the LoCal+SGD algorithms achieve upto ~1.4× reduction in runtime compared to the baseline, while sacrificing <0.5% loss in Top-1 accuracy. The static LoCal+SGD algorithm exhibits slightly superior runtime performance for similar accuracies compared to the dynamic algorithm. However, as noted earlier, the dynamic algorithm eliminates the effort required to identify an optimal scheduling function.

**Table 1:** ImageNet.

**Network**	**Training strategy**	**Top-1 error (%)**	**Speed-up**
ResNet34	Baseline SGD	26.6	1×
	LoCal+SGD (Static)	**27**	**1.34×**
	LoCal+SGD (Dynamic)	**27.04**	**1.26×**
	Training with Stochastic depth	27.89	1.13×
	Freezing layers during training	27.32	1.36×
ResNet50	Baseline SGD	24.02	1×
	LoCal+SGD (Static)	**24.51**	**1.42×**
	LoCal+SGD (Dynamic)	**24.45**	**1.37×**
	Training with Stochastic depth	26.76	1.08×
	Pruning during training	24.89	1.32×
	Freezing layers during training	24.84	1.49×
MobileNetV2	Baseline SGD	28.41	1×
	LoCal+SGD (Static)	**28.90**	**1.32×**
	LoCal+SGD (Dynamic)	**28.98**	**1.27×**
	Training with Stochastic depth	30.53	1.17×
	Freezing layers during training	29.31	1.54×

*All experimental results pertaining to LoCal+SGD are highlighted in bold*.

Table 1 also compares the performance of LoCal+SGD against existing research efforts designed to improve training efficiency. We perform this analysis against two efforts, namely (i) Training with stochastic depth (Huang et al., 2016) and (ii) Structured Pruning during Training (Lym et al., 2019).

Training with stochastic depth, as the name suggests, stochastically bypasses residual blocks by propagating input activations/error gradients via identity or downsampling transformations, resulting in improved training time. However, the approach is targeted toward extremely deep networks and as seen in Table 1, it incurs a noticeable accuracy loss on networks such as ResNet34, ResNet50 and MobileNetV2. Compared to training with stochastic depth, our proposal clearly achieves better accuracy as well as training runtime benefits. The key principle behind the pruning during training approach is to reduce the size of the weight and activation tensors in a structured manner during training, thereby providing speed-ups on GPU/TPU platforms. However, on complex benchmarks such as ResNet50, such techniques achieve speed-ups at the cost of significant drop in accuracy (~1.5%). To further demonstrate the utility of localized updates in our approach, we consider a third technique, wherein layers selected to be updated locally for a given epoch are instead frozen, i.e., the parameters are held fixed during that epoch. While this achieves better runtime savings, it incurs considerably higher loss in accuracy, further underscoring the benefits of LoCal+SGD.

**Figure 5:**
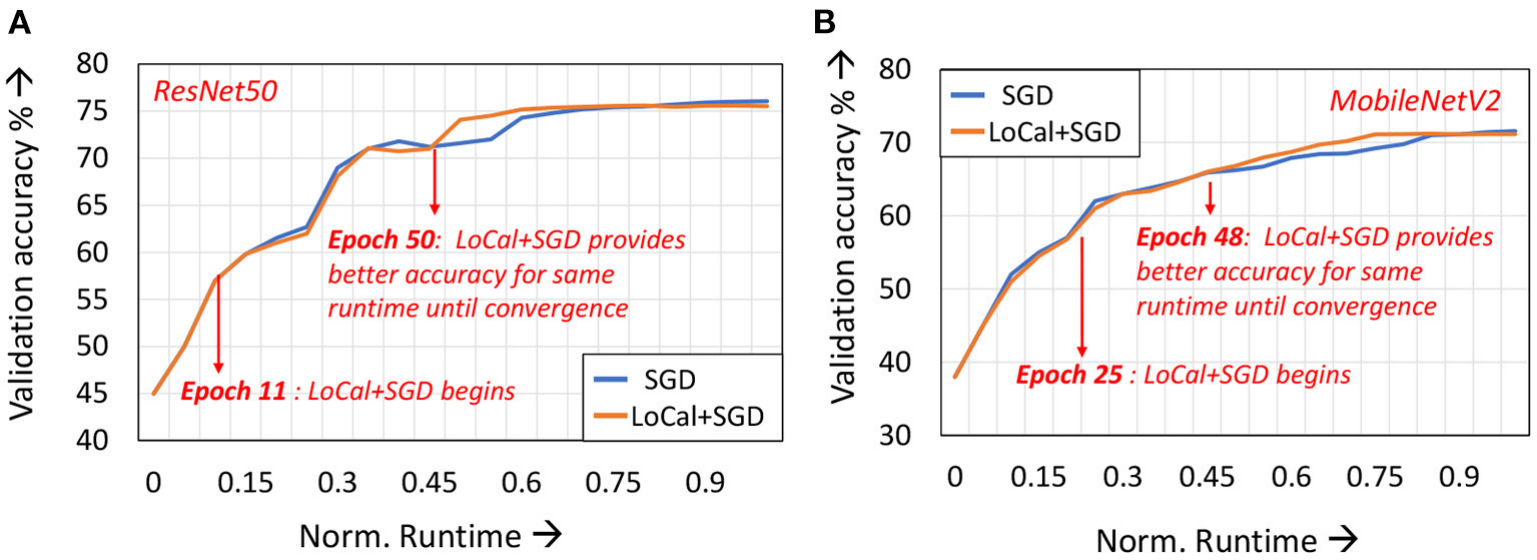
Validation accuracies across training runtime for **(A)** ResNet50 and **(B)** MobileNetV2.

In Figure 5, we depict the validation accuracy curves for the ResNet50 and MobileNetV2 benchmarks trained with LoCal+SGD and SGD, normalized to SGD training runtime. For the sake of brevity, we have presented the curves when using the dynamic learning mode selection algorithm. As can be seen, after a few epochs have passed since localized updates began, LoCal+SGD achieves better accuracies for the same runtime.

**CIFAR-10 and CIFAR-100:** Table 2 presents the accuracy and corresponding compute benefits of the baseline and the proposed technique, as well as training with stochastic depth and layer freezing, for the CIFAR-10 and CIFAR-100 datasets. Stochastic depth is applicable only to residual blocks and is hence not considered for the VGG-13 network. Across benchmarks, we observe upto a 1.51× improvement in training runtime. Compared to the ImageNet benchmarks, LoCal+SGD applies localized updates more aggressively in the CIFAR-10 and CIFAR-100 benchmarks i.e., more layers are updated locally for a higher number of epochs. This leads to superior compute benefits on these benchmarks.

**Table 2:** Cifar10 and Cifar100.

**Network (Dataset)**	**Training strategy**	**Top-1 err. (%)**	**Speed-up**
ResNet18	Baseline SGD	6.06	1×
(Cifar10)	LoCal+SGD (Static)	**6.17**	**1.53×**
	LoCal+SGD (Dynamic)	**6.23**	**1.43×**
	Training with Stochastic depth	6.79	1.35×
	Freezing layers during training	6.51	1.65×
VGG13 (Cifar10)	Baseline SGD	7.16	1×
	LoCal+SGD (Static)	**7.28**	**1.32×**
	LoCal+SGD (Dynamic)	**7.25**	**1.28×**
	Freezing layers during training	7.43	1.42×
ResNet18 (Cifar100)	Baseline SGD	23.39	1×
	LoCal+SGD (Static)	**23.61**	**1.47×**
	LoCal+SGD (Dynamic)	**23.63**	**1.44×**
	Training with Stochastic depth	23.97	1.35×
	Freezing layers during training	23.74	1.62×
VGG13 (Cifar100)	Baseline SGD	31.36	1×
	LoCal+SGD (Static)	**31.56**	**1.3×**
	LoCal+SGD (Dynamic)	**31.59**	**1.32×**
	Freezing layers during training	31.94	1.42×

*All experimental results pertaining to LoCal+SGD are highlighted in bold*.

In Table 3 we compare the final accuracy obtained by LoCal+SGD against the baseline for the same time budget across all our benchmarks. We note that the time budget considered is the time taken by LoCal+SGD to complete all epochs of training. Clearly, within the same time budget LoCal+SGD achieves better accuracy than baseline SGD.

**Table 3:** Comparing accuracy at Iso-runtime.

**Dataset**	**Network**	**Top-1 err. with LoCal+SGD (%)**	**Top-1 err. with baseline SGD (%)**
ImageNet	ResNet34	27.04	27.36
	ResNet50	24.41	24.67
	MobileNetV2	28.94	29.18
Cifar10	VGG13	7.25	7.56
	ResNet18	6.23	6.47
Cifar100	VGG13	31.59	31.9
	ResNet18	23.63	23.97

### 3.3. Execution Time Benefits for Multi-GPU Training

**Figure 6:**
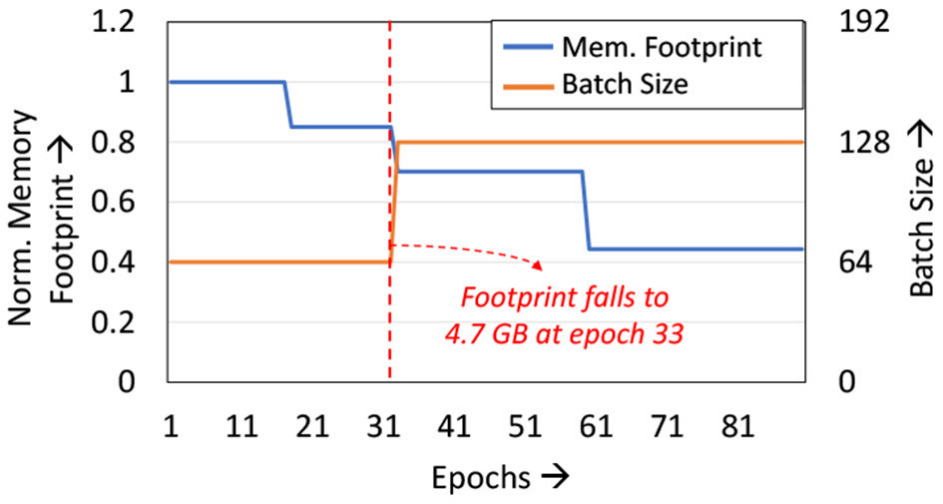
Analyzing memory footprint and batch-size variation.

We analyze the memory footprint of the ResNet50 network when trained with LoCal+SGD on the ImageNet dataset (Figure 6). Training first commences with all layers updated with SGD, resulting in a high memory footprint. Due to the 10 GB capacity of the chosen GPU, the mini-batch size is limited to 64 per GPU. As the *Localized*→*SGD* transition layer progresses across the network, the memory footprint required also gradually reduces across epochs. We take advantage of this reduction in memory footprint in the context of distributed training using 4 GPUs with data parallelism. Specifically, we extract additional runtime benefits by increasing the batch size on each GPU, which reduces the frequency of gradient aggregation between devices and alleviates the communication overhead. At epoch 33, the memory footprint per GPU reduces to <5 GB, allowing training with an increased mini-batch size of 128 per GPU from epoch 33 onwards. As seen in Table 4, the doubling of the batch-size provides an additional 6% improvement in total training time. We note that other training techniques such as training with stochastic depth cannot exploit this feature, since they do not impact memory footprint substantially.

**Table 4:** Analyzing impact of increasing batch-size on ImageNet.

**Network**	**Training strategy**	**Top-1 err. (%)**	**Speed-up**
ResNet50	Baseline SGD (fixed batch-size)	24.06	1×
	LoCal+SGD (fixed batch-size)	**24.48**	**1.27×**
	LoCal+SGD (variable batch-size)	**24.51**	**1.34×**

*All experimental results pertaining to LoCal+SGD are highlighted in bold*.

### 3.4. Visualizing Activation Distributions

**Figure 7:**
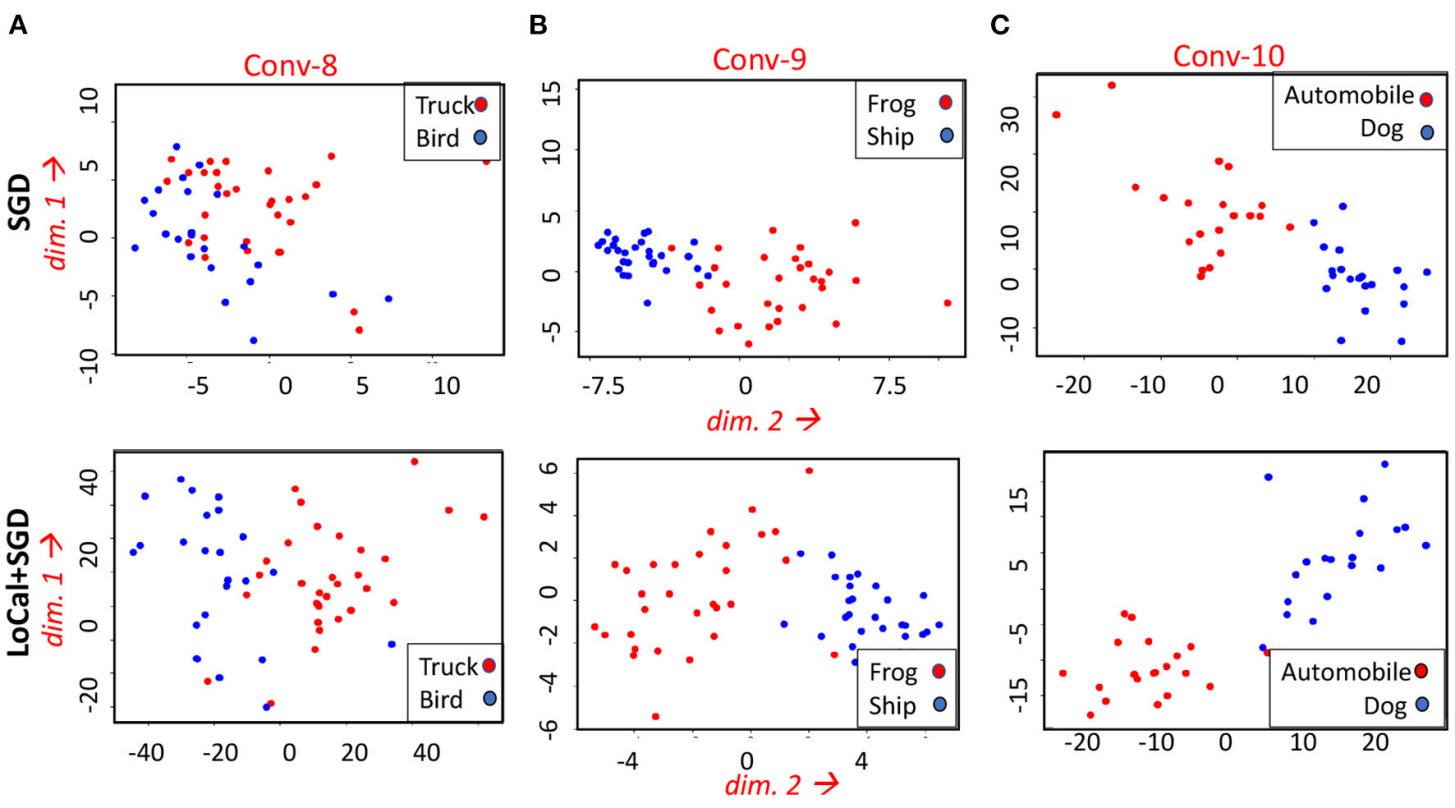
Comparing the top 2 principal components at different layers of the ResNet18-Cifar10 benchmark for the following classes **(A)** Truck and Bird **(B)** Frog and Ship **(C)** Automobile and Dog.

**Figure 8:**
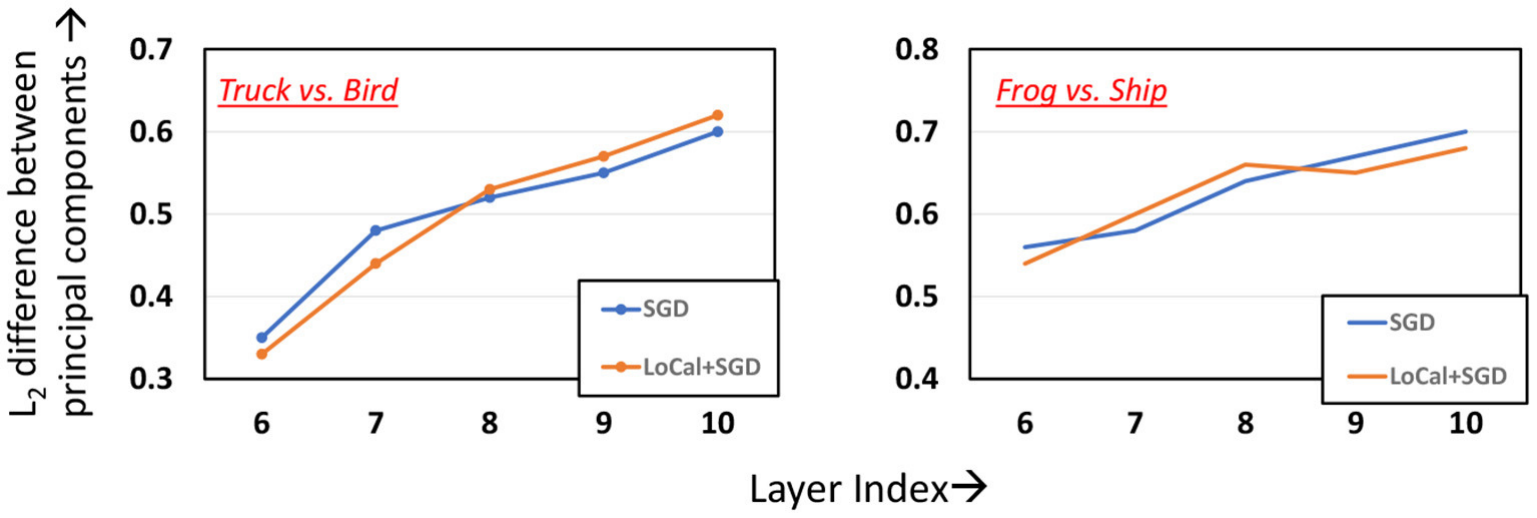
Analyzing *L*_2_ difference between the principal components across the layers.

LoCal+SGD utilizes localized updates to adjust the weights of the initial feature-extraction layers. As discussed previously, these localized updates have been demonstrated to approximate popular unsupervised learning algorithms such as principal component analysis, k-means clustering, *etc*. We illustrate this in the context of LoCal+SGD. To this end, after training is complete, we extract the top 2 principal components of the activation outputs of the locally updated layers. For comparison, this procedure is repeated for the same layers when they are updated with SGD instead. In Figure 7, we have plotted the dominant components of activations of the second and fourth convolutional layers of the ResNet18-Cifar10 benchmark, when trained with SGD and LoCal+SGD. Interestingly, we find that LoCal+SGD provides comparable (Figures 7B,C), or in some cases even better separation (Figure 7A) between the classes compared to SGD. We illustrate this further in Figure 8, wherein we plot the *L*_2_ difference between the top-2 principal components of either class across selected layers of the network. It is noteworthy that LoCal+SGD achieves these separations while requiring a substantially lower number of operations per convolutional layer.

### 3.5. Ablation Studies

As mentioned in section 2, the efficacy of the static and dynamic learning mode selection algorithms are controlled by different hyper-parameters. The performance of the static selection algorithm is dictated by *c*_1_ and *c*_2_, while α and *L*_*shift*_ impact the dynamic algorithm. Different values of these parameters can result in different learning mode configurations during training, resulting in different points in the computational efficiency vs. accuracy trade-off space. To understand this trade-off, we individually study the impact of each parameter. Further, we also discuss the impact of the weak supervision scheme on accuracy.

We begin by first analyzing the impact of the α and *L*_*shift*_ parameters used in the dynamic learning mode selection algorithm.

**Figure 9:**
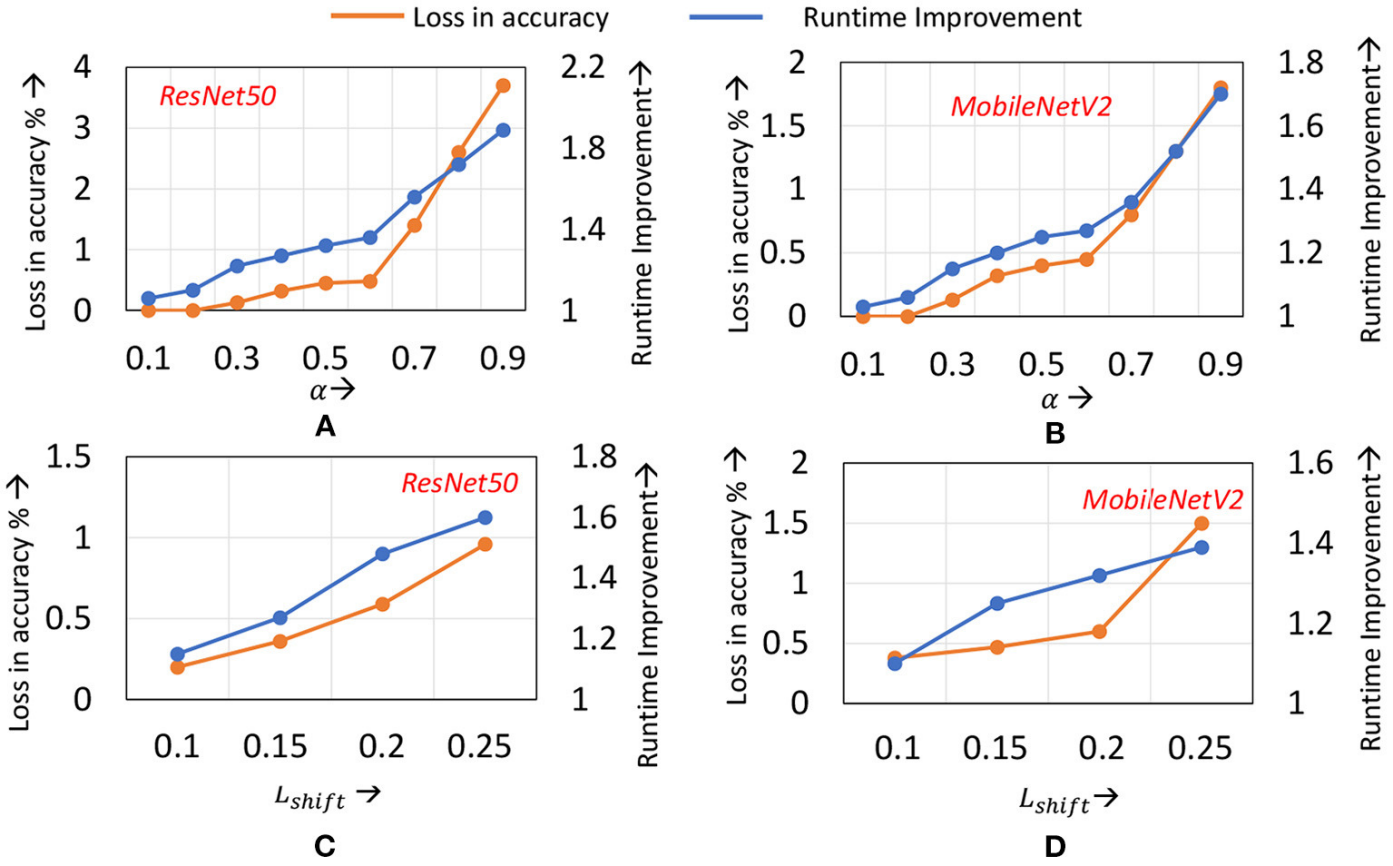
Compute efficiency vs. accuracy trade-off on ImageNet when **(A)** α is varied for ResNet50 **(B)** α is varied for MobileNetV2 **(C)** *L*_*shift*_ is varied for ResNet50 **(D)** *L*_*shift*_ is varied for MobileNetV2.

*Impact of* α: Figures 9A,B illustrate the runtime savings and accuracy achieved for different values of α, for the ResNet50 and MobileNetV2 benchmarks on ImageNet. For both benchmarks, increasing α from 0.1 until 0.5 improves the runtime benefits as the application of localized updates increases, while maintaining the loss in accuracy to within 0.2–0.3%. However, once α exceeds 0.6, the degradations in accuracy exceed 0.5% on both benchmarks. The speedups increase to 1.6× when around 1% loss in accuracy is tolerable.

*Impact of* *L*_*shift*_: In Figures 9C,D we highlight the impact of different *L*_*shift*_ values (recall that *L*_*shift*_ denotes the amount by which we shift the transition layer). Note that we have normalized *L*_*shift*_ to the total network depth. The graphs indicate that for *L*_*shift*_ values between 10 and 15% of the total number of layers in the network, the loss in accuracy remains within 0.5%, and the runtime savings increase with increasing *L*_*shift*_. However, when *L*_*shift*_ exceeds 15%, the accuracy begins to degrade. This can be attributed to the simultaneous transition in the learning mode of a large number of layers affecting convergence of training.

From Figure 9, we make an additional observation—across ResNet50 to MobileNetV2, similar values of α and *L*_*shift*_ provide a good trade-off. We find that this observation holds for other ImageNet benchmarks analyzed, such as ResNet34. We therefore utilize a common set of hyper-parameter values for all networks of a particular dataset. This eliminates the need to conduct a hyper-parameter search process to determine α and *L*_*shift*_ for every new network that is to be trained.

**Figure 10:**
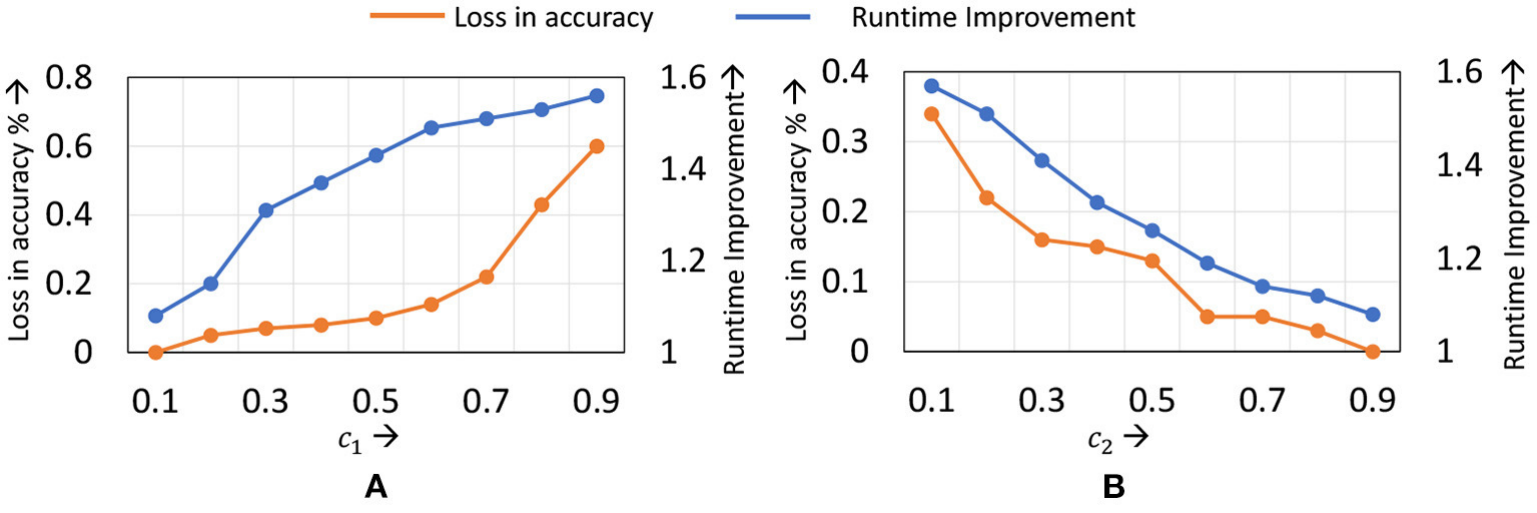
Compute efficiency *vs*. accuracy trade-off obtained by varying **(A)** *c*_1_ and **(B)** *c*_2_ in the static learning mode selection algorithm for ResNet18 on the Cifar10 dataset.

The static learning mode selection algorithm is controlled by two parameters *c*_1_ and *c*_2_. *c*_1_ represents the maximum number of layers to which localized updates are applies, while *c*_2_ controls the epoch at which localized updates begin. In Figure 10, we present the accuracy and runtime benefits obtained when varying these parameters for the ResNet18 network on the Cifar10 dataset. *c*_1_ is represented as a fraction of the total number of layers in the network, while *c*_2_ is expressed as a fraction of the total training epochs. We observe that to achieve a good trade-off, setting *c*_1_ between 0.5 and 0.7, and *c*_2_ in the range of 0.2–0.3 provides best results. As with the dynamic learning mode selection algorithm, we find that common *c*_1_ and *c*_2_ values can be used for all networks of a particular dataset, with only marginal impact on performance.

*Impact of weak supervision*: In Table 5, we highlight the impact of the weak supervision technique on final classification accuracy. Across all our benchmarks, the weak supervision technique improves accuracy by 0.06–0.17%, bringing the final accuracy of LoCal+SGD closer to baseline SGD. This improvement comes at no cost in runtime, since the overhead of modulating the learning rate of locally updated layers is negligible.

**Table 5:** Impact of weak supervision on accuracy.

**Dataset**	**Network**	**Top-1 err. with weak supervision (%)**	**Top-1 err. without weak supervision (%)**
ImageNet	ResNet34	27.04	27.1
	ResNet50	24.41	24.49
	MobileNetV2	28.94	29.03
Cifar10	VGG13	7.25	7.39
	ResNet18	6.23	6.41
Cifar100	VGG13	31.59	31.7
	ResNet18	23.63	23.75

### 3.6. LoCal+Adam

We analyze the impact of combining localized learning with other gradient descent based learning algorithms such as Adam. In Table 6, we successfully demonstrate LoCal+Adam on the Cifar10-ResNet18 benchmark. We note that all other aspects of the design such as the learning mode selection algorithm etc., remain unchanged. These results thus speak to the widespread applicability of our technique, irrespective of the gradient descent learning algorithm used.

**Table 6:** Accuracy and runtime benefits of LoCal+Adam.

**Training technique**	**Top-1 error (%)**.	**Speed-up**
Adam	7.7	1×
LoCal+Adam	7.96	1.48×

### 3.7. Applicability of LoCal+SGD to Other Networks

In this paper, LoCal+SGD has been explored and demonstrated with a focus on convolutional neural networks. We demonstrate the applicability of LoCal+SGD to segmentation networks such as U-Net (Ronneberger et al., 2015). The long-range connections in U-Net are handled similar to the shortcut connections in ResNets. Consider a Layer K, whose input and output activations are *A*_*K*−1_ and *A*_*K*_. Further, let us assume Layer K receives activation input *A*_*J*_ from a preceding layer J. The weight update for Layer K is performed by convolving the summed activation *A*_*K*_ + *A*_*J*_, with *A*_*K*−1_. Table 7 demonstrates the applicability of LoCal+SGD to U-Net training on the ISBI 2012 challenge dataset (Arganda-Carreras et al., 2015).

**Table 7:** Accuracy and runtime benefits of LoCal+SGD on U-Net.

**Training technique**	**Dice coefficient**.	**Speed-up**
Baseline SGD	0.948	1×
LoCal+SGD	0.943	1.26×

## 4. Related Work

This section discusses research directions that are related to LoCal+SGD. These efforts can be broadly categorized into two classes. The first class of efforts focus on improving the computational efficiency of gradient-descent based DNN training. The second class of efforts involve the design of neuro-inspired learning rules such as feedback alignment, *etc*. (Nøkland, 2016). Our work is orthogonal to both classes of efforts, since our focus is on how to selectively combine localized learning rules with SGD for better computational efficiency. In section 3, we demonstrated how LoCal+SGD achieves superior accuracy vs. computational efficiency trade-off than some of these efforts. We next elaborate upon the research efforts in both aforementioned directions.

**Hyper-parameter tuning:** Many efforts are directed toward achieving training efficiency by controlling the hyper-parameters involved in gradient-descent, notably the learning rate. For example (Akiba et al., 2017; Goyal et al., 2017; You et al., 2017a,b) propose learning rate tuning algorithms that accelerate training with no loss in accuracy, and scale to hundreds of CPU/GPU cores.

**Model size reduction during training:** Model size reduction via pruning and quantization is a popular technique to reduce compute costs during inference. In many of these efforts, a dense or full precision model is re-trained or fine-tuned to obtain a pruned or quantized model. However, recent efforts have also investigated dynamically pruning (Lym et al., 2019) or quantizing (Sun et al., 2019) a model during training itself, resulting in training speed-ups. Taking a slightly different approach (Huang et al., 2016) proposes stochastically dropping residual blocks on extremely deep networks such as ResNet-1202, not only for training runtime benefits but also better accuracies due to improved gradient strength.

**Instance importance based training:** Recent research efforts have discovered that not all training samples are required for improving loss minimization during SGD training (Jiang et al., 2019; Zhang et al., 2019). That is, a sizable fraction of the samples can be skipped during several epochs, depending on their impact on the classification loss evaluated during *FP*. This translates to a reduction in mini-batches per epoch, providing considerable runtime benefits.

**Neuro-inspired learning rules:** Back-propagation algorithms utilized in DNN training are not biologically plausible, i.e., they greatly differ from how learning happens in the brain. To this end, there have been several efforts that propose biologically plausible learning algorithms. These algorithms have demonstrated considerable success on complex networks and datasets. For example, feedback alignmnent algorithms (Nøkland, 2016) tackle the weight transport problem (Liao et al., 2015) by allowing for asymmetry in the weight values during forward and back propagation. Likewise, target propagation (Lee et al., 2015) encourages neural activity to reach desired target activations evaluated during forward propagation, instead of utilizing loss gradients. In equilibrium propagation (Scellier and Bengio, 2017), the gradients of locally defined objective functions are used to update the weights of a layer, thereby eliminating the propagation of gradients across the network.

LoCal+SGD represents a new direction wherein we combine localized learning and SGD in the context of an overall supervised learning framework, with the goal of reducing training time. Therefore, we surmise that advances in either SGD-based learning or localized learning can be incorporated within LoCal+SGD to further advance its benefits.

## 5. Conclusion

In this paper, we introduce a new approach to improve the training efficiency of state-of-the-art DNNs. Specifically, we take advantage of the computationally efficient nature of localized learning rules and selectively update some layers with these rules instead of SGD. We design a learning mode selection algorithm that determines the learning mode for the layers of the network in every epoch in order to achieve a favorable tradoff between training time and accuracy. Further, we also implement a low-cost weak supervision scheme that brings the accuracy of the proposed scheme closer to traditional SGD-based training. Across a benchmark suite of 8 DNNs, we achieve upto 1.5× reduction in training times on a modern GPU platform.

## Data Availability Statement

The original contributions presented in the study are included in the article/supplementary material, further inquiries can be directed to the corresponding author/s.

## Author Contributions

SK devised and conducted experiments. All authors contributed to the formulation of the problem statement.

## Funding

This work was supported in part by Semiconductor Research Corporation (SRC).

## Conflict of Interest

SS and SV are employed by IBM Research. The remaining authors declare that the research was conducted in the absence of any commercial or financial relationships that could be construed as a potential conflict of interest.

## Publisher's Note

All claims expressed in this article are solely those of the authors and do not necessarily represent those of their affiliated organizations, or those of the publisher, the editors and the reviewers. Any product that may be evaluated in this article, or claim that may be made by its manufacturer, is not guaranteed or endorsed by the publisher.

## Footnotes

^1^In addition to combining localized and SGD based learning, LoCal+SGD is Low-Calorie SGD or SGD with reduced computational requirements.
